# Application of λ esophagojejunostomy in total gastrectomy under laparoscopy: a modified technique for post-gastrectomy reconstruction

**DOI:** 10.3389/fonc.2024.1335297

**Published:** 2024-08-09

**Authors:** Lang-Biao Liu, Guo-Tian Ruan, Ya-Dong Wu, Lei Niu, Jun Cai

**Affiliations:** ^1^ Department of General Surgery, Beijing Friendship Hospital, Capital Medical University, Beijing, China; ^2^ State Key Lab of Digestive Health, National Clinical Research Center for Digestive Diseases, Beijing, China

**Keywords:** laparoscopy, total gastrectomy, lambda anastomosis, Roux-en-Y anastomosis, non-dismembered Roux-en-Y anastomosis

## Abstract

**Objective:**

Common gastrectomy methods can significantly affect patients’ postoperative quality of life. This study investigated the safety, feasibility, and short-term efficacy of λ-type esophagojejunostomy in total gastrectomy under total laparoscopy.

**Methods:**

We retrospectively analyzed the clinical and follow-up data of 50 patients with adenocarcinoma of the gastric/gastroesophageal junction who underwent total laparoscopic radical gastrectomy with λ-type esophagojejunostomy at the Beijing Friendship Hospital from January 2021 to July 2022. Data are reported as mean ± standard deviation.

**Results:**

Patients comprised 27 males and 23 females, aged 42 to 76 (60.9 ± 5.6) years. There were 26 cases of gastroesophageal junction adenocarcinoma (16 Siewert type II and 10 Siewert type III) and 24 cases of adenocarcinoma of the proximal gastric body. All patients underwent radical total gastrectomy and D2 lymph node dissection with λ-type esophagojejunostomy for digestive tract reconstruction under total laparoscopy. The total operation time was 235–295 (249.4 ± 48.5) min, digestive tract reconstruction time was (48.2 ± 23.2) min, intraoperative blood loss was (63.4 ± 48.4) mL, recovery time of exhaust was (3.1 ± 2.2) d, first drinking or eating time was (4.1 ± 2.1) d, and hospital stay was (9.3 ± 4.4) d. Three patients had postoperative complications, including one with duodenal remnant leakage combined with abdominal infection. Anastomotic bleeding and postoperative inflammatory intestinal obstruction occurred in one patient each, all of whom were cured by conservative treatment. The Nutritional Risk Index of the whole group was 53.5 ± 8.4 preoperatively, 47.3 ± 5.6 one week postoperatively, 50.3 ± 5.6 six months postoperatively, and 52.4 ± 4.2 at 12 months postoperatively. Roux-en-Y stasis syndrome and bile reflux esophagitis occurred in one patient each (2.0%). There were no occurrences of recanalization of the closed end of the afferent loop of the esophagojejunostomy anastomosis, anastomotic stricture or obstruction, or tumor recurrence.

**Conclusion:**

λ-type esophagojejunostomy is safe and feasible for digestive tract reconstruction after total laparoscopic radical gastrectomy. This digestive tract reconstruction method not only maintains intestinal continuity but also simplifies surgical procedures, allowing patients to recover quickly with an excellent short-term effect.

## 1 Introduction

With the increasing maturity of laparoscopic technology and the rapid evolution of laparoscopic instruments, laparoscopic radical gastrectomy for gastric cancer is being performed in surgical centers across China. Owing to an increasing incidence of gastric cancer in the upper and middle parts of the stomach (1–4), the proportion of total gastrectomies performed laparoscopically is also increasing. However, surgical reconstruction of the digestive tract after total gastrectomy, particularly esophagojejunostomy, is challenging in clinical practice (5–7). The anastomosis should not only ensure smoothness and adequate blood flow but also reduce the tension of the anastomotic mouth. Roux-en-Y anastomosis is the most commonly used anastomosis method in total gastrectomy; however, even in the absence of obstructive factors, postoperative symptoms such as chronic abdominal pain, persistent nausea, intermittent vomiting, and weight loss may arise. Collectively referred to as Roux stasis syndrome, these symptoms can significantly affect patients’ postoperative quality of life (8–11). Although a new, non-interrupted method of Roux-en-Y digestive tract reconstruction after distal gastrectomy has been proposed (12, 13), its safety and feasibility are not yet well established (14–17). Our hospital has developed several improvements to this process to overcome the main disadvantages of the traditional reconstruction method. Under full laparoscopy, the self-traction technique is used to free the lower esophagus, providing a better view and operational space for anastomosis. Cutting the mesentery significantly reduces tension at the anastomosis mouth, thereby ensuring adequate blood flow. The anastomosis is completed using a laparoscopic linear stapler, and the digestive tract was then reconstructed using a non-interrupted Roux-en-Y anastomosis. The shape of the post-anastomosis esophagojejunostomy is similar to the Greek letter “λ“ (lambda), thus prompting us to refer to it as a λ esophagojejunostomy. Herein, we report the results of our study exploring the safety, feasibility, and efficacy of this new method of digestive tract reconstruction after total gastrectomy.

## 2 Materials and methods

### 2.1 General information

Retrospective data were available for patients with gastric cancer who underwent total gastrectomy under laparoscopy using the λ esophagojejunostomy at the Beijing Friendship Hospital, Capital Medical University, from January 2021 to July 2022. The inclusion criteria were as follows: 1). Diagnosis of gastric adenocarcinoma through gastroscopy confirmed via pathology; 2). Tumor center located at the esophagogastric junction (Siewert types II–III), the gastric fundus, proximal stomach body, or involving the entire stomach; 3). Preoperatively assessed as eligible for R0 curative total gastrectomy; 4). Patient provision of informed consent; 5). Successful D2 lymph node dissection and intracavitary λ esophagojejunostomy for digestive tract reconstruction; 6). Surgery completed by the same surgical team without transition to open surgery. 7). Complete follow-up data through July 2023. The exclusion criteria were as follows: 1). Pre-gastrectomy neoadjuvant therapy or palliative surgical treatment; 2). Distant metastases or involvement of surrounding organs; 3). Emergency surgery owing to tumor rupture causing major bleeding, perforation, or obstruction. After implementing the inclusion and exclusion criteria, 50 patients with gastric cancer were included in the study.

### 2.2 Surgical procedure

Patients were intubated and placed under general anesthesia, assuming a supine position with the legs spread, head elevated, and feet lowered by 10–15°C. Preserving aesthetics around the navel, a 10 mm vertical incision was made close to the upper edge of the navel, followed by the establishment of CO_2_ pneumoperitoneum with pressure set to 10–12 mmHg (1 mmHg = 0.133 kPa). A V-shaped 5-port layout was used. The surgeon stood on the patient’s right side, and the assistant, on the patient’s left side, had the camera operator between the legs. After setting up the trocar, a routine laparoscopic examination was performed for distant metastases, and the left liver lobe was suspended (18). D2 lymph node dissection was performed according to the Japanese Gastric Cancer Treatment Guidelines, 5^th^ edition (19).

After transecting the duodenum with a linear stapler, the proximal 2–3 cm margin near the His angle was determined. In cases involving the esophagus, intraoperative gastroscopy was performed to locate the upper edge of the tumor precisely, and a resection line 2–3 cm proximal to it was marked. The lower esophagus was ligated and tractioned with a sterile strap, allowing for “self-traction” while the subdiaphragmatic clearance and the mobilization of the lower esophagus were completed. A hole was made in the posterior right wall of the esophagus proximal to the traction string and resection line for anastomosis preparation. The jejunum, approximately 25–30 cm distal to the ligament of Treitz, was brought up to the left side of the esophagus, with the mesentery at peak tension. An ultrasonic scalpel was used to carve out the mesentery and sever one to three primary vessels from the mesenteric artery. Preserving the marginal arcades of the jejunum is crucial during this procedure. Once sufficient tension was released from the jejunal loop, a hole was created at the mesenteric edge of the bowel in its most relaxed position. With the surgeon’s left-hand instrument pulling the traction cord toward the splenic pole, the assistant used a linear stapler longer than 45 mm to perform side-to-side esophagojejunostomy by inserting the cartridge and anvil into holes made in the jejunum and esophagus, respectively. The common opening was then closed, the esophagus was transected using a stapler, and the specimen was removed through a 3–5 cm vertical auxiliary incision at the navel.

**Figure 1:**
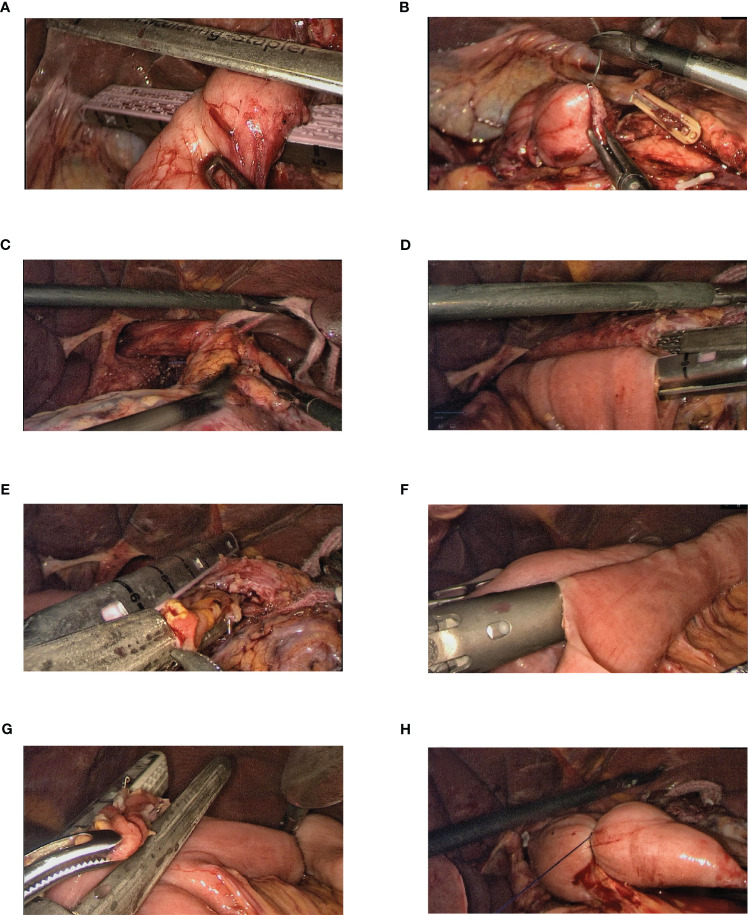
Steps of the full surgical procedure. **(A, B)** The duodenum is transected using a linear stapler, and the remnant of the duodenum is sutured and buried. **(C)** With the esophagus in a “self-traction” state, the lower mediastinal clearance is completed and the lower segment of the esophagus is mobilized. **(D)** A hole is made in the right posterior wall of the esophagus proximal to the marked resection line. The jejunum, approximately 25–30 cm distal to the ligament of Treitz, is brought up, and when sufficiently tension-free, a hole is created at its mesenteric edge. Laparoscopic side-to-side esophagojejunostomy was performed using a linear stapler. **(E)** Using a linear stapler, the common opening was closed while simultaneously transecting the lower segment of the esophagus. **(F)** Holes are created in the mesenteric edges of the jejunal loops, 10 cm from the ligament of Treitz (input loop) and 40 cm from the esophagojejunal anastomosis (output loop). A side-to-side Braun anastomosis was then performed between these jejunal loops. **(G)** The common opening of the Braun anastomosis was closed using a linear stapler. **(H)** A double purse-string suture is made with a 3-0 Prolene thread 3 cm from the esophagojejunal anastomosis on the input loop, thereby occluding the jejunum.

**Figure 2:**
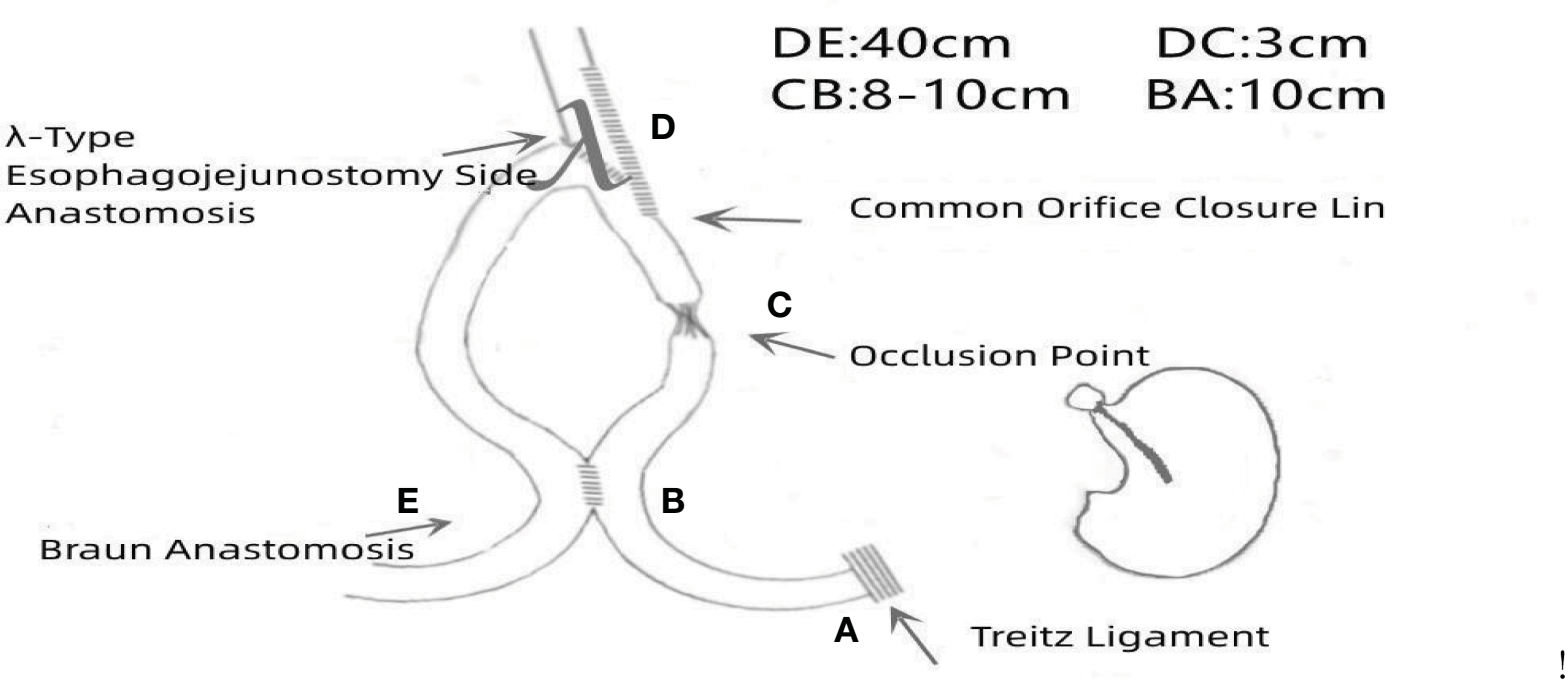
Schematic illustration of the surgical procedure.

Upon re-establishing the pneumoperitoneum, holes were made in the mesenteric edges of the jejunal loops, 10 cm from the ligament of Treitz (input loop) and 40 cm from the esophagojejunal anastomosis (output loop). Side-to-side Braun anastomosis was performed between the jejunal loops. The common opening was closed using the stapler 3 cm from the esophagojejunal anastomosis on the input loop, and a double purse-string suture was made with 3-0 Prolene thread, completing the non-disconnected Roux-en-Y anastomotic digestive tract reconstruction. The full surgical procedure is depicted in **Figure 1**, and a schematic illustration is shown in **Figure 2**.

### 2.3 Observation indicators and follow-up

The parameters under observation included total surgery time, time taken for gastrointestinal reconstruction, intraoperative blood loss, and postoperative gas passage recovery time. Other factors recorded were the post-surgery times for the first intake of water or food and the duration of postoperative hospitalization. Postoperative complications (e.g., anastomotic leakage, postoperative abdominal infection, anastomotic hemorrhage, postoperative pneumonia, pleural effusion, and wound infections) were monitored. Patients were followed up through phone calls and outpatient visits at one week and post-discharge months six and 12 months post-discharge. Follow-up indicators included endoscopy and gastrointestinal imaging (assessing the anastomosis for recurrence, stenosis, and patency of the closed segment of the input jejunal loop), bile reflux esophagitis, Roux-en-Y retention syndrome (chronic upper abdominal pain, postprandial fullness, nausea, and intermittent vomiting), and postoperative nutritional status. The Nutritional Risk Index (PNI) was defined as serum albumin (g/L) +5 × lymphocyte count × 10^9^/L (7). Tumor recurrence and metastasis were also assessed during follow-up. The cut-off date for follow-up was July 7, 2023.

### 2.4 Statistical analysis

Continuous variables satisfying a normal distribution were reported as mean ± standard deviation. Continuous variables that did not conform to the normal distribution are represented by the median (interquartile range). Categorical variables were reported as numbers and percentages.

## 3 Results

### 3.1 Patient characteristics and intraoperative and postoperative outcomes

The average age of the patients was 60.9 ± 5.6 years, with 27 males (54%) and 23 females (46%). There were 26 cases of adenocarcinoma of the gastroesophageal junction (16 cases of Siewert type II and 10 cases of Siewert type III) and 24 cases of adenocarcinoma of the proximal gastric body. The patients’ demographic and clinical characteristics are shown in **Table 1**.

**Table 1:** General information of 50 patients undergoing radical total gastrectomy for gastric cancer.

Variables	λ Esophagojejunostomy (n=50)	Percent(%)
Gender		
Female/Male	27/23	54/46
Age (years)		
≤60/>60	19/31	38/62
ASA		
I-II/III	17/33	15/85
Location of tumor		
Gastric fundus and Gastric body/Esophagog-astric junction	24/26	48/52
Pathological T stage		
T1-T2/T3-T4	4/46	8/92
Pathological LN metastasis		
N0/N+	11/39	22/78
Pathological TNM		
I+II/III	4/46	8/92
Differentiation degree		
High/Medium-Low/Low	2/18/30	4/36/60

Percent(%) were used to analyze the basic characteristics. ASA, American Society of Anesthesiologists. LN, Lymph node.

Total surgery time: 249.4 ± 48.5 min; time for gastrointestinal reconstruction: 48.2 ± 23.2 min; intraoperative blood loss: 63.4 ± 48.4 mL; time to postoperative gas passage recovery: 3.1 ± 2.2 d; time to first intake of water or food post-surgery: 4.1 ± 2.1 d; and duration of postoperative hospitalization: 9.3 ± 4.4 d. Three patients experienced postoperative complications, specifically leakage from the duodenal stump accompanied by abdominal infection, hemorrhage from the anastomotic site, and postoperative inflammatory bowel obstruction. All patients were treated conservatively and recovered. No complication-related fatalities were observed. The details of the patient outcomes are presented in **Table 2**.

**Table 2:** Surgery and postoperative details of undergoing radical total gastrectomy for patients with gastric cancer.

Variables	λ Esophagojejunostomy (n=50)
Operative time, min (mean (SD))	249.4 ± 48.5
Intraoperative anastomosis time, min (mean (SD))	48.2 ± 23.2
Intraoperative blood loss, ml, (mean (SD))	63.4 ± 48.4
Length of hospital stay, days (mean (SD))	9.3 ± 4.4
postoperative gas passage recovery, days (mean (SD))	3.1 ± 2.2
Intake time of liquid food, days (mean (SD))	4.1 ± 2.1
Overall short-term postoperative complications, N (%)	3(6)
Serious complications (Clavien III-V), N (%)	1(2)
Anastomotic Hemorrhage, N (%)	1(2)
Inflammatory Bowel Obstruction, N (%)	1(2)
Roux-en-Y Stasis Syndrome, N (%)	1(2)
Bile Reflux Esophagitis, N (%)	1(2)
Nutritional indicator (PNI)	
Pre-surgery (mean (SD))	53.5 ± 8.4
1 week post-surgery (mean (SD))	47.3 ± 5.6
6 months post-surgery (mean (SD))	50.3 ± 5.6
12 months post-surgery (mean (SD))	52.4 ± 4.2

SD, standard deviation; N, number; PNI, Prognostic nutritional index.

### 3.2 Follow-up results

**Figure 3:**
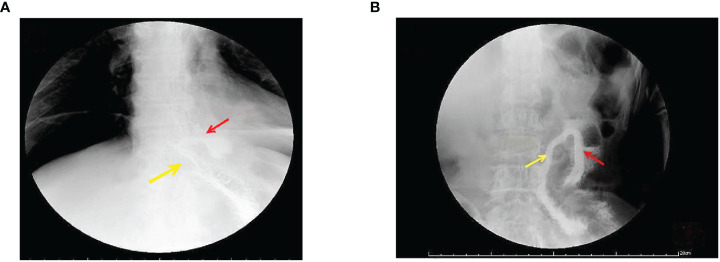
Gastrointestinal angiography at 1 week postoperative. **(A)** Radiographic examination of the digestive tract 1 week postoperatively. The yellow arrow indicates a patent outflow loop at the esophagojejunal anastomosis, and the red arrow points to the non-dissected closed blind end, showing no recanalization. **(B)** Radiographic examination of the digestive tract 12 months postoperatively. The yellow arrow indicates a patent outflow loop at the esophago-jejunal anastomosis, and the red arrow points to the non-dissected closed blind end, showing no recanalization.

**Figure 4:**
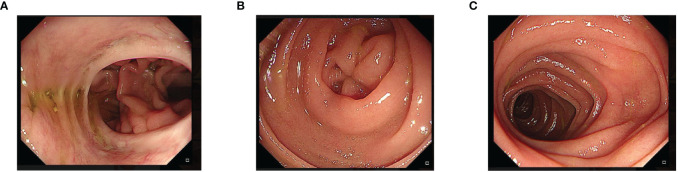
Endoscopic examination at 12 months postoperative. A patent esophagojejunal anastomosis can be seen, without signs of stenosis or recurrence at the anastomotic site. **(A)** One year later, endoscopic esophagojejunum showed no tumor recurrence and stricture. **(B)** One year later, the blind end of the endoscope was closed and no recanalization was found. **(C)** One year later, the jejunum of the output loop of endoscope was unobstructed.

Comprehensive follow-up data were obtained for all 20 patients beginning 1 week postoperatively. Postoperative nutritional indicators were recorded at one week, 6 months, and 12 months. The overall PNI results were as follows: preoperative: 53.5 ± 8.4; 1 week postoperative: 47.3 ± 5.6; 6 months postoperative: 50.3 ± 5.6; 12 months postoperative: 52.4 ± 4.2; weight loss at 12 months postoperative was 2.0 ± 3.2 kg. Only one patient (2.0%) presented with moderate-to-severe anemia at 12 months postoperatively. Roux-en-Y retention syndrome and bile reflux esophagitis each occurred in one patient (2.0%). Endoscopic examinations and upper gastrointestinal imaging conducted one week and 12 months postoperatively revealed no tumor recurrence at the esophagojejunal anastomotic site nor any narrowing or obstruction. The detailed results are presented in **Table 2** and **Figures 3**, **4**.

## 4 Discussion

Reconstruction of the digestive tract after laparoscopic radical total gastrectomy poses a significant challenge. The quest among gastrointestinal surgeons has been to identify a surgical technique that is straightforward and effective and ensures rapid postoperative recovery with minimal complications. We use esophageal self-traction technology under the whole abdominal cavity through innovation to obtain a better esophageal area operation space and free esophageal length. This laparoscopic approach allowed efficient transection and tension reduction of the jejunal mesentery, whereas the linear cutter stapler facilitated side-to-side anastomosis of the esophagus and jejunum with mechanical closure of the shared opening. Subsequently, the digestive tract was reconstructed using the Uncut Roux-en-Y technique (12, 13).

During surgery, the esophagogastric junction was sterilized and pulled caudally to maximize the visibility of the distal esophagus and its surrounding tissues. This not only ensured a longer resectable esophageal distance under laparoscopy but also guaranteed a thorough lymph node dissection, a safe distance from the proximal resection margin, and a more spacious operative field for subsequent esophagojejunal anastomosis. Compared with the traditional Roux-en-Y anastomosis; after adopting esophageal self-traction technology, we have more obvious advantages in the surgical field of vision and full exposure of the esophageal area. Compared to the traditional Roux-en-Y match, the most challenging aspect of side-to-side esophagojejunal ((-type) anastomosis is the release of match tension. We innovated again based on the distribution of the small mesenteric blood supply, selectively ligating one to three blood vessels of the mesenteric artery’s first-level longitudinal branch to relieve tension. This enabled the ligation of a greater number of first-level branches, ensuring a bidirectional blood supply to the anastomosis site from the marginal arcade and avoiding jejunal loop ischemia, tension-free anastomosis, and lower anastomotic leak rates.

When performing esophagojejunal anastomosis, placing the input loop on the left side is recommended, as the suture line for closing the common opening is also located on the left side, falling over the input loop and negating concerns about potential stricture. During anastomosis, laparoscopic linear cutter staplers 45 or 60 mm in length were used, ensuring a side-to-side anastomosis stitch length ≥45 mm, resulting in an anastomotic opening diameter of approximately 3 cm. This ensured the patency of the anastomotic site. After completing the side-to-side anastomosis, the stapler was used to dissect the esophagus and close the shared opening simultaneously, simplifying the procedure and reducing the overall digestive tract reconstruction time (20, 21) and, thereby, the surgery cost. This study’s mean digestive tract reconstruction time was 48.2 ± 23.2 min, closely aligning with previous literature (22). The mean intraoperative blood loss amounted to 63.4 ± 48.4 mL; postoperative recovery to first flatus took 3.1 ± 2.2 d, and the initiation of drinking or eating was 4.1 ± 2.1 d; the overall hospital stay was 9.3 ± 4.4 d. After completing the jejunal Braun anastomosis, we ligated the input jejunal loop 3 cm from the esophagojejunal anastomosis using 3-0 silk sutures. This ligation aimed to prevent bile and pancreatic juice reflux, thereby preventing reflux esophagitis. The ligation point was carefully positioned 3 cm away from the anastomotic site to avoid anastomotic constriction or ischemia if placed too close and to preclude food stagnation and, thus, potential blind loop obstruction if positioned too far away. Tension on the ligation suture was adjusted to ensure complete bowel closure and prevent bile and pancreatic juice reflux without inducing ischemic necrosis. Although there have been reports of successful closure using staplers (16), the process requires specialized equipment and is relatively expensive. Concerns have been raised in the English literature (23) about the potential reopening of the obstructed jejunal loop during Uncut Roux-en-Y anastomosis, leading to severe reflux esophagitis and prompting subsequent Roux-en-Y anastomosis. However, using double ligation with 3-0 silk sutures to obstruct the input jejunal segment successfully prevented re-openings visible on endoscopy or gastrointestinal radiography during the 12-month postoperative follow-up period.

Our postoperative complication rate was an admirable 6% (3/50)— with one case each of duodenal stump leak with intra-abdominal infection, anastomotic bleeding, and postoperative inflammatory bowel obstruction. This aligns closely with the results of previous reports (24, 25). Of particular note was a patient with anastomotic bleeding that occurred 48 h postoperatively, manifesting as fresh blood from the gastric tube accompanied by a small amount of melena. The patient showed a progressive decrease in hemoglobin levels that stabilized after multiple transfusions, repeated gastric tube irrigation with cold saline, and administration of hemostatic medications. All other postoperative complications were managed conservatively and resolved, with no mortality-associated events. At the 1-year postoperative follow-up, most patients had body weights and PNI values close to their preoperative values. This suggests that our digestive tract reconstruction technique after total gastrectomy is effective, with patients displaying good postoperative digestive and absorptive function.

When closing the shared opening and dissecting the specimen, we avoided cutting the jejunum and mesentery. However, traditional Roux-en-Y anastomosis requires cutting off the intestine, disrupting the continuity of the jejunum. We could not ensure that there were no aberrations in the pacing potentials of the gut that affected electrical conduction during intestinal peristalsis. Our matching method can also prevent reflux of alkaline substances such as bile, pancreatic juice, and intestinal fluid, reducing the rates of reflux esophagitis and rapid gastric emptying syndrome (RGE) (10). In our study, RGE syndrome was observed in only one patient (2%). Some researchers have proposed that combining Uncut Roux-en-Y anastomosis with anterior colonic anastomosis can substantially reduce the risk of internal hernias (12, 22). As a result, we did not close Peterson’s space, and no internal hernias were observed during patient follow-up.

This study has several limitations. Specifically, our results may reflect a relatively small cohort size and a short postoperative follow-up period. Additionally, we lack comparative data for patients who underwent conventional Roux-en-Y anastomosis for postgastrectomy reconstruction.

## 5 Conclusion

Our study found that combining post-self-traction esophageal dissection and side-to-side esophagojejunal λ-type anastomosis with the Uncut Roux-en-Y technique is safe, feasible, and effective. However, a thorough understanding of the indications for anastomosis and the technique itself is crucial. A randomized controlled trial with a larger patient cohort is needed to compare the results of our method with those of conventional Roux-en-Y anastomosis.

## Data Availability

The raw data supporting the conclusions of this article will be made available by the authors, without undue reservation.
